# Haploinsufficiency of *ETV6* and *CDKN1B* in patients with acute myeloid leukemia and complex karyotype

**DOI:** 10.1186/1471-2164-15-784

**Published:** 2014-09-11

**Authors:** Simone Feurstein, Frank G Rücker, Lars Bullinger, Winfried Hofmann, Georgi Manukjan, Gudrun Göhring, Ulrich Lehmann, Michael Heuser, Arnold Ganser, Konstanze Döhner, Brigitte Schlegelberger, Doris Steinemann

**Affiliations:** Institute of Cell and Molecular Pathology, Hannover Medical School, Hannover, Germany; Department of Internal Medicine III, University Hospital of Ulm, Ulm, Germany; Institute of Pathology, Hannover Medical School, Hannover, Germany; Department of Hematology, Hemostasis, Oncology, and Stem Cell Transplantation, Hannover Medical School, Hannover, Germany

**Keywords:** Acute myeloid leukemia (AML), Complex karyotype, Haploinsufficiency, *ETV6*, *CDKN1B*, ArrayCGH, Methylation, Gene expression

## Abstract

**Background:**

Acute myeloid leukemia with complex karyotype (CK-AML) is a distinct biological entity associated with a very poor outcome. Since complex karyotypes frequently contain deletions of the chromosomal region 12p13 encompassing the tumor suppressor genes *ETV6* and *CDKN1B,* we aimed to unravel their modes of inactivation in CK-AML.

**Results:**

To decipher deletions, mutations and methylation of *ETV6* and *CDKN1B*, arrayCGH, SNP arrays, direct sequencing of all coding exons and pyrosequencing of the 5′UTR CpG islands of *ETV6* and *CDKN1B* were performed. In total, 39 of 79 patients (49%) showed monoallelic deletions of 12p13 according to karyotypic data and 20 of 43 patients (47%) according to genomic profiling. Genomic profiling led to the minimal deleted region covering the 3′-UTR of *ETV6* and *CDKN1B*. Direct sequencing revealed one novel monoallelic frameshift mutation in *ETV6* while no mutations in *CDKN1B* were identified. Furthermore, methylation levels of *ETV6* and *CDKN1B* did not indicate transcriptional silencing of any of these genes. *ETV6* and *CDKN1B* had reduced expression levels in CK-AML patients with deletion in 12p13 as compared to CK-AML without deletion in 12p13, while the other genes (*BCL2L14, LRP6, DUSP16* and *GPRC5D)* located within the minimal deleted region in 12p13 had very low or missing expression in CK-AML irrespective of their copy number status.

**Conclusions:**

*ETV6* and *CDKN1B* are mainly affected by small monoallelic deletions, whereas mutations and hypermethylation play a minor role in CK-AML. Reduced gene dosage led to reduced gene expression levels, pointing to haploinsufficiency as the relevant mechanism of inactivation of *ETV6* and *CDKN1B* in CK-AML.

**Electronic supplementary material:**

The online version of this article (doi:10.1186/1471-2164-15-784) contains supplementary material, which is available to authorized users.

## Background

Acute myeloid leukemia (AML) is a hematopoietic malignancy of clonal myeloid progenitor cells arrested at an immature differentiation stage. There is substantial phenotypic and genetic heterogeneity due to the acquisition of different genetic and/or epigenetic alterations in leukemia-initiating cells [1]. Acute myeloid leukemia with complex karyotype (CK-AML) is a distinct biological entity, traditionally defined by the presence of at least three independent chromosome aberrations, excluding t(8;21), inv(16)/t(16;16), and t(15;17) and is associated with a very poor outcome [2, 3]. More than 150 genes have been shown to be differentially expressed in CK-AML compared to AML with normal karyotype, including several genes located on 5q and 7q as well as genes involved in DNA repair, chromosome segregation, and within the actin cytoskeleton [4]. CK-AML often contains deletions of 5q, 7q, and 17p and shows high incidence of somatic alterations of *TP53* [5–7].

Besides these characteristic deletions, complex karyotypes frequently contain deletions of the chromosomal region 12p13 [8–10]. The reported minimal deleted region of 12p13 spans two putative tumor suppressor genes, *ETV6* and *CDKN1B* [11–13]. For both genes, haploinsufficiency as the underlying mechanism was previously suggested, as they are located within the minimal deleted region and inactivation of the second allele is rarely seen [12, 14, 15]. Furthermore, haploinsufficiency of *CDKN1B* is strongly implicated in numerous cancer types, the vast majority of lymphatic origin [16, 17].

*ETV6 (ets translocation variant gene 6),* a member of the ETS transcription factor family, shows several properties of a putative tumor suppressor gene like induction of G1 arrest and blocking of Ras-induced transformation [18], induction of apoptosis [19], and activation of *TP53* dependent pathways [20]. *CDKN1B* encodes the p27CDKN1B protein which belongs to the CIP/KIP class of cyclin dependent kinase inhibitors and inactivates the cyclin E/CDK2 complex via binding of CDKN1B to cyclin E/CDK2 [21]. *CDKN1B* is hence an important negative regulator of the cell cycle.

In this study, we aimed to investigate whether and how *ETV6* and *CDKN1B* are inactivated by (small) deletions, mutations or DNA methylation in the specific subgroup of CK-AML.

## Results

### Standard karyotyping and genomic profiling of 12p13

We extensively characterized a cohort of 79 patients with CK-AML. Thirty-nine of them (49%) showed a loss of 12p13 according to karyotyping (Additional file 1: Table S1). This included deletions of 12p13 due to interstitial deletions, unbalanced translocations, and monosomy 12. A monosomy 12 was observed in 13 patients, a deletion of 12p13 either by interstitial or terminal deletion or by additive chromosomal material in 21 patients and a dicentric or derivative chromosome with loss of 12p in five patients. The frequency of cytogenetically detectable -5/5q-, -7/7q- or -17/17p- did not differ in the groups of CK-AML with and without 12p13 deletion (analyzed with Fisher’s exact test, p-values: -5/5q- 0.81, -7/7q- 0.17, -17/17p- 0.36).

**Figure 1:**
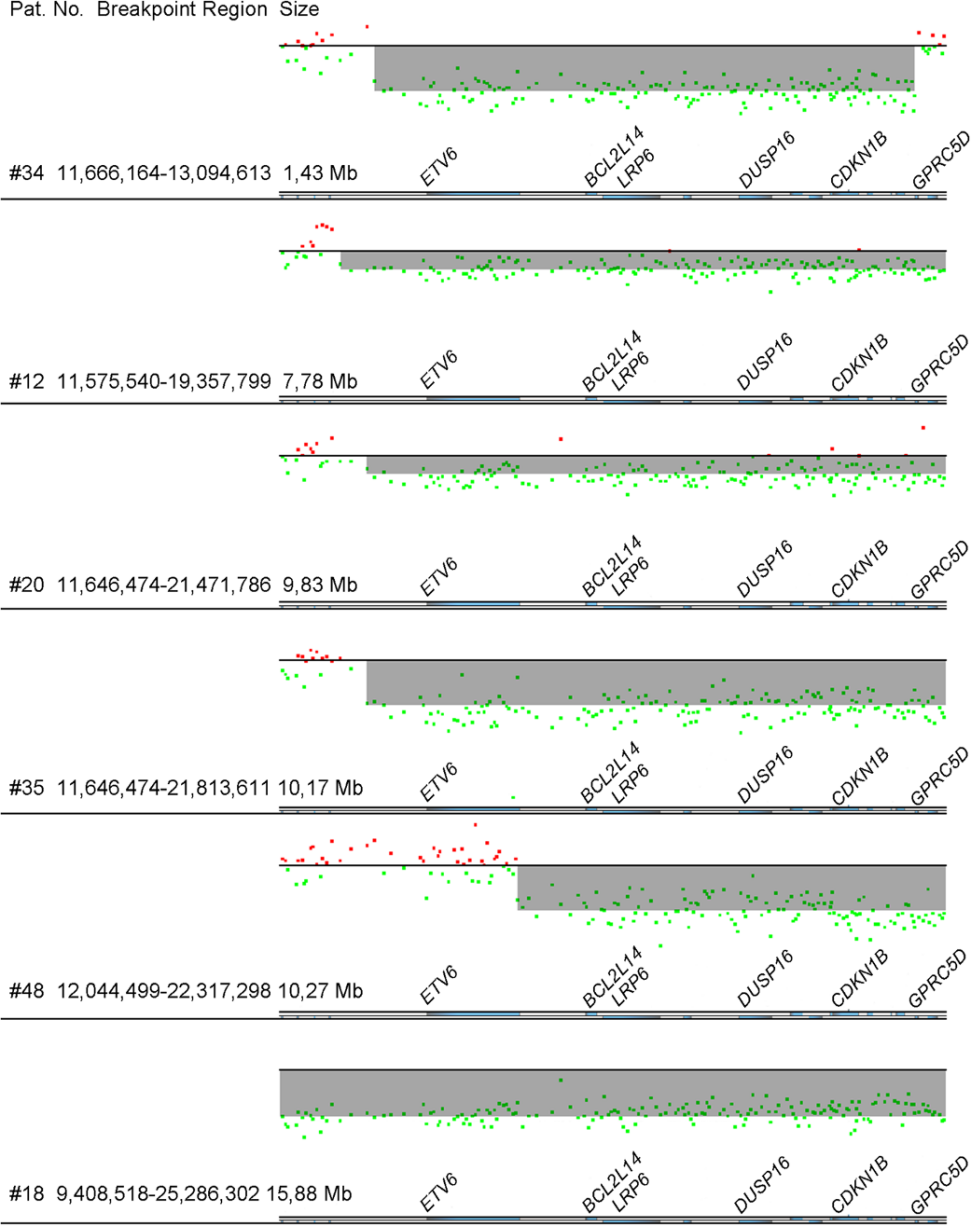
**Interstitial 12p13 deletions.** ArrayCGH results (244 k, Agilent) showing interstitial 12p13 deletions in six patients. The genomic profiles are zoomed in to the minimal deleted region covering *ETV6* at the telomeric site and *CDKN1B* at the centromeric site. Deleted regions are highlighted in gray. Next to the breakpoint region given in Mb, the size of the deletion is shown. The *ETV6* gene is orientated in 5′-3′ direction. Mean log ratios of the deletions correlate with the clone size according to karyotyping.

In 43 patients, DNA was available for genomic profiling [array comparative genomic hybridization (arrayCGH) or single-nucleotide polymorphism (SNP) arrays] to determine the allelic status of 12p13 and delineate the breakpoints of 12p more closely. In 26 of these patients we used arrayCGH methods (244 k array, 2.8 k array and 8.0 k array), five patients were analyzed by using the SNP 250 k array and 12 patients were analyzed by using the SNP 6.0 array. In summary, in 20 of 43 patients (47%), losses of 12p13 could be identified by genomic profiling encompassing the *ETV6* and *CDKN1B* genes. With this approach, three patients were newly detected to carry a deletion in 12p13 (#61, #64, #77), however in six patients (#13, #22, #36, #41, #69, #70) a loss of 12p13 according to karyotyping was not confirmed (Additional file 1: Table S1). Small interstitial deletions within the chromosomal region 12p13 were identified in 13 of 43 patients with CK-AML analyzed by arrayCGH and SNP arrays. The distal breakpoints mapped within a region of 30 kb directly 5′ to *ETV6*, except in one patient (#48), where the first deleted probe was localized in the 3′UTR of *ETV6.* The proximal breakpoints were all downstream of *GPRC5D* (G-protein-coupled receptor, family C, group 5). The minimal deleted region in our cohort spanned 1.43 Mb and included *CDKN1B* and the 3′UTR of *ETV6* (Figure 1).

### 12p13 deletion breakpoints

**Figure 2:**
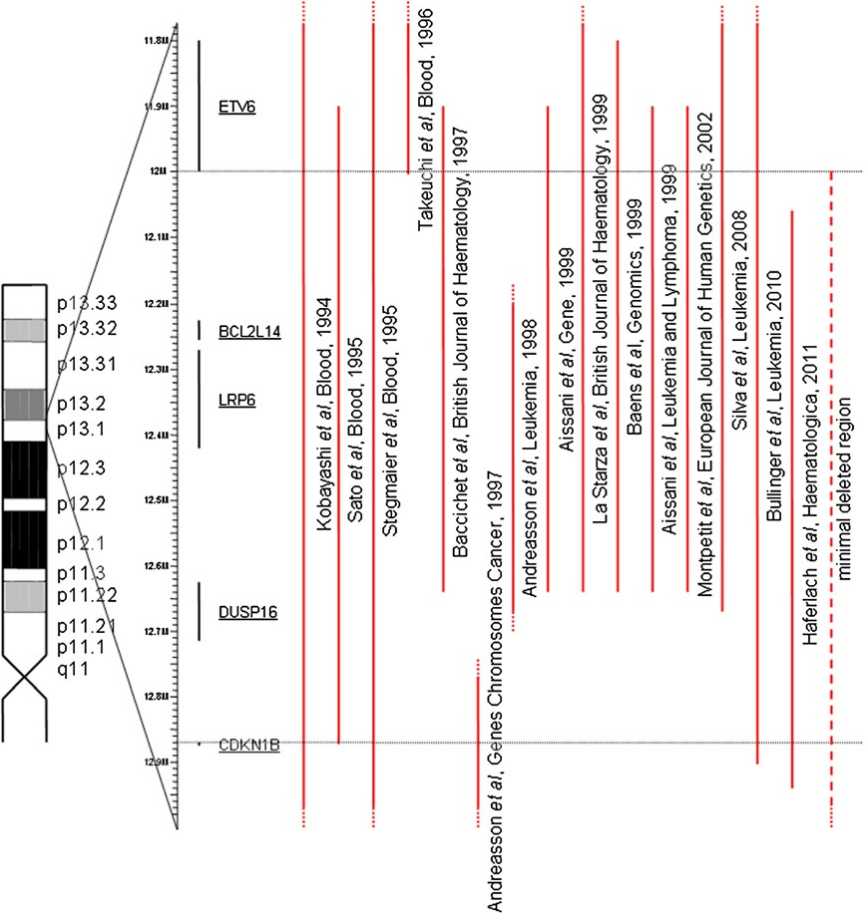
**12p minimal deleted regions from different studies.** The ideogram of the chromosome arm 12p is shown on the left. The region from 11.8 Mb to 12.9 Mb is zoomed in to the genes *ETV6, BCL2L14, LRP6, DUSP16*, and *CDKN1B* given as black bars. The red bars to the right of the genes indicate the minimal deleted regions as described in the different studies shown below. Most of these studies refer to (CK-) AML or contain a large number of patients with AML. The red bar on the right displays the minimal deleted region identified in this study. A dashed line indicates that the border of the deletion is not exactly determined or exceeds the selected chromosomal region.

A graphic overview of the 12p13 deletion breakpoints including or excluding *ETV6* and *CDKN1B* from different studies also comprising our data is shown in Figure 2. Most of these studies refer to (CK-) AML or contain a large number of patients with AML. Some studies reported minimal deleted regions containing either *ETV6* or *CDKN1B*. However, in the majority of studies, the minimal deleted region covered both *ETV6* and *CDKN1B*.

### Mutation analyses of *ETV6, CDKN1B*and *TP53*

**Figure 3:**
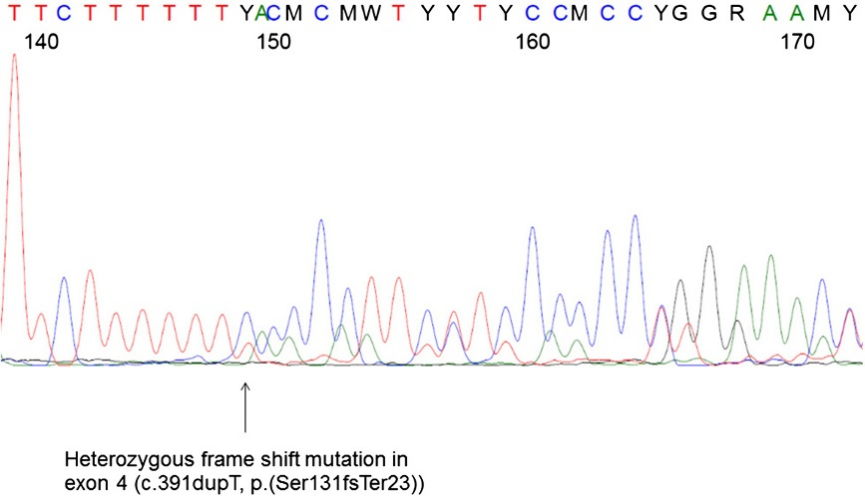
***ETV6*** **mutation.** Heterozygous frame shift mutation in exon 4, c.391dupT, p.(Ser131PhefsTer23), leading to truncated protein due to a newly generated stop codon (patient #21).

To further determine the modes of inactivation of *ETV6* [NM_001987] and *CDKN1B* [NM_004064], we performed mutation analyses of all coding exons of *ETV6* in 56 patients and of *CDKN1B* in 67 patients with and without 12p13 deletions for which DNA was available (Additional file 1: Table S1). In our cohort of CK-AML, no *CDKN1B* mutations were identified. However, in *ETV6* we found a distinct and novel heterozygous frameshift mutation of exon 4, c.391dupT, p.(Ser131PhefsTer23) in one patient (#21) (Figure 3). This frameshift mutation lies within the N-terminal homodimerization domain and theoretically would disturb homodimerization, thus leading to a complete loss of the DNA-binding ETS domain. A deleterious effect was confirmed by using the platform PROVEAN for non-synonymous or indel variants and prediction of functional effects (http://provean.jcvi.org/index.php) [22]. The mutation was detected at the time point of relapse. Importantly, in the first diagnostic sample of this patient still showing a normal karyotype, the *ETV6* mutation was present, indicating that this mutation emerged early in the course of the disease. In the following samples during the course of the disease, a complex karyotype with several balanced translocations and a terminal deletion of 14q was detected (Additional file 1: Table S1). By means of arrayCGH, no gains or losses in the region of the *ETV6* gene were evident. Notably, no *TP53, FLT3* nor *CEBPA* mutations (data not shown) and no typical cytogenetic aberrations of CK-AML like del(5q), -7/del(7q), or del(17p) were present in this patient. However an *NPM1* mutation (c.860_863dup, p.(Trp288CysfsTer12)) was identified (data not shown).

To determine the frequency of *TP53* mutations in the subgroup with and without 12p13 deletions, we performed mutation analysis of all coding exons of *TP53* in all 79 patients. In 44 of 79 patients (56%), *TP53* mutations were identified (see Additional file 2: Table S2). The frequency of 12p13 alterations was similar in patients with and without a monoallelic or biallelic inactivation of *TP53* (45.5% versus 45.7%, p = 0.99).

### Methylation analyses of *ETV6*and *CDKN1B*

**Figure 4:**
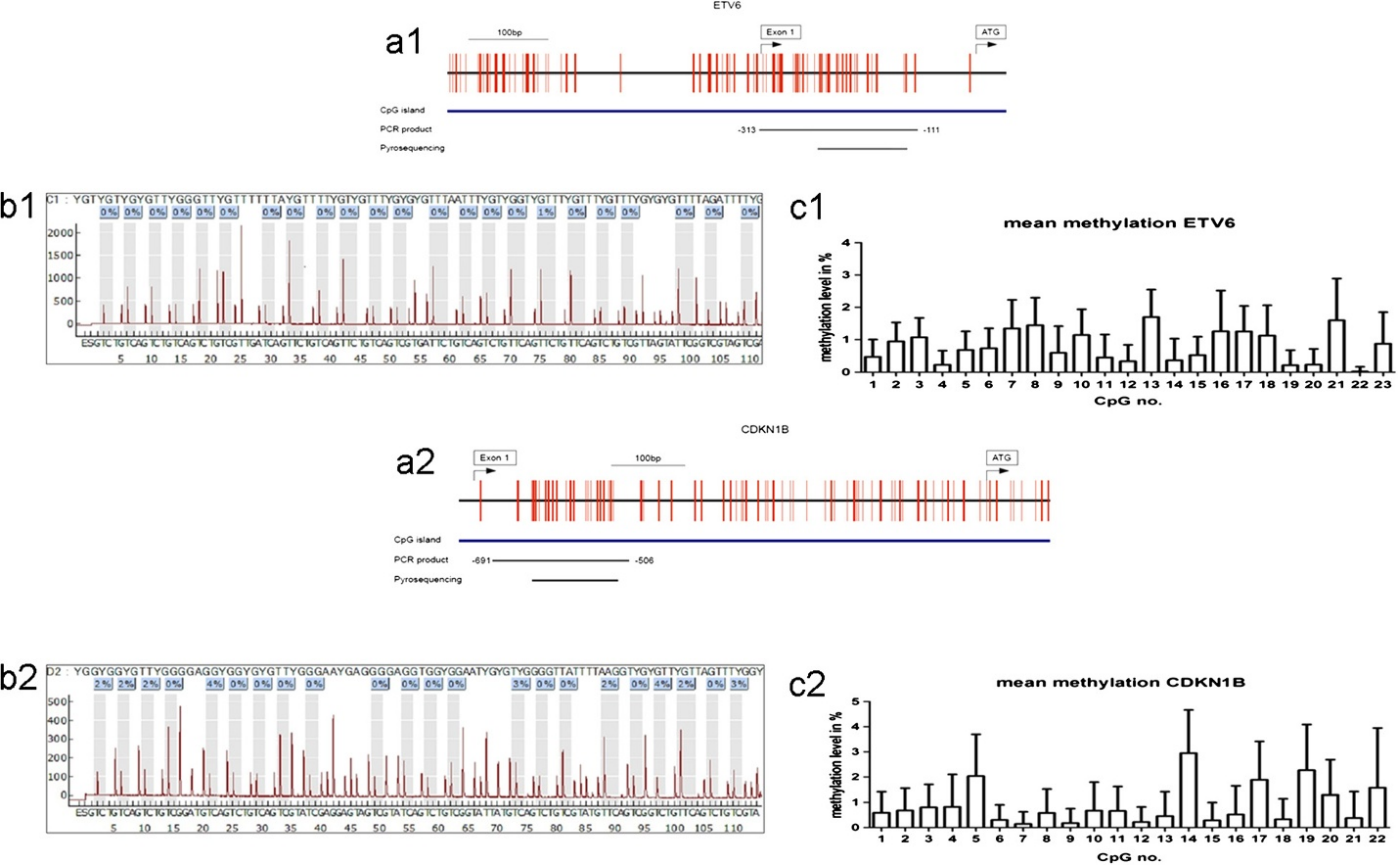
**Methylation analyses of** ***ETV6*** **and** ***CDKN1B*** **. a1/a2:** Schematic view of the *ETV6/CDKN1B*-CpG islands: each vertical bar represents a single CpG site. Arrows indicate the start of the non-coding exon 1 and the start codon. The positions of the PCR product and the product used for pyrosequencing are indicated below as horizontal bars. **b1/b2:** A representative pyrogram determining the nucleotide sequence within the CpG island of *ETV6/CDKN1B*. The sequence in the upper part of the pyrogram contains a Y at positions under investigation, the sequence below indicates the injected bases during pyrosequencing. Highlighted areas in the pyrogram indicate variable CpG positions (light gray). The methylation level of each CpG site is indicated in blue boxes on top of the pyrogram. **c1/c2:** Mean methylation levels of the 23 CpG sites in the 5′UTR of *ETV6* and the 22 CpG sites in the 5′UTR of *CDKN1B* of all patients analyzed are shown as a percentage. The standard deviation was determined using the Kruskal-Wallis test.

To determine the methylation status of *ETV6* and *CDKN1B*, quantitative methylation analysis of the 5′UTRs of both genes was performed by pyrosequencing. We examined 23 single CpG sites within the *ETV6* 5′UTR CpG island and 22 single CpG sites within the *CDKN1B* 5′UTR CpG island in 55 patients (27 with 12p13 deletions, 28 without 12p13 deletions). The mean methylation levels of *ETV6* and *CDKN1B* ranged from 0% to 1.43% and 0.09% to 1.77%, respectively (Figure 4, Additional file 1: Table S1). These results did not exceed the cut-off levels of 5% indicative for increased methylation. Neither did the methylation degree of single CpG sites in the patients exceed the cut-off level. In summary, no evidence of increased methylation of the 5′UTR of either gene was found.

### Expression profiling of the genes within the minimal deleted region

**Figure 5:**
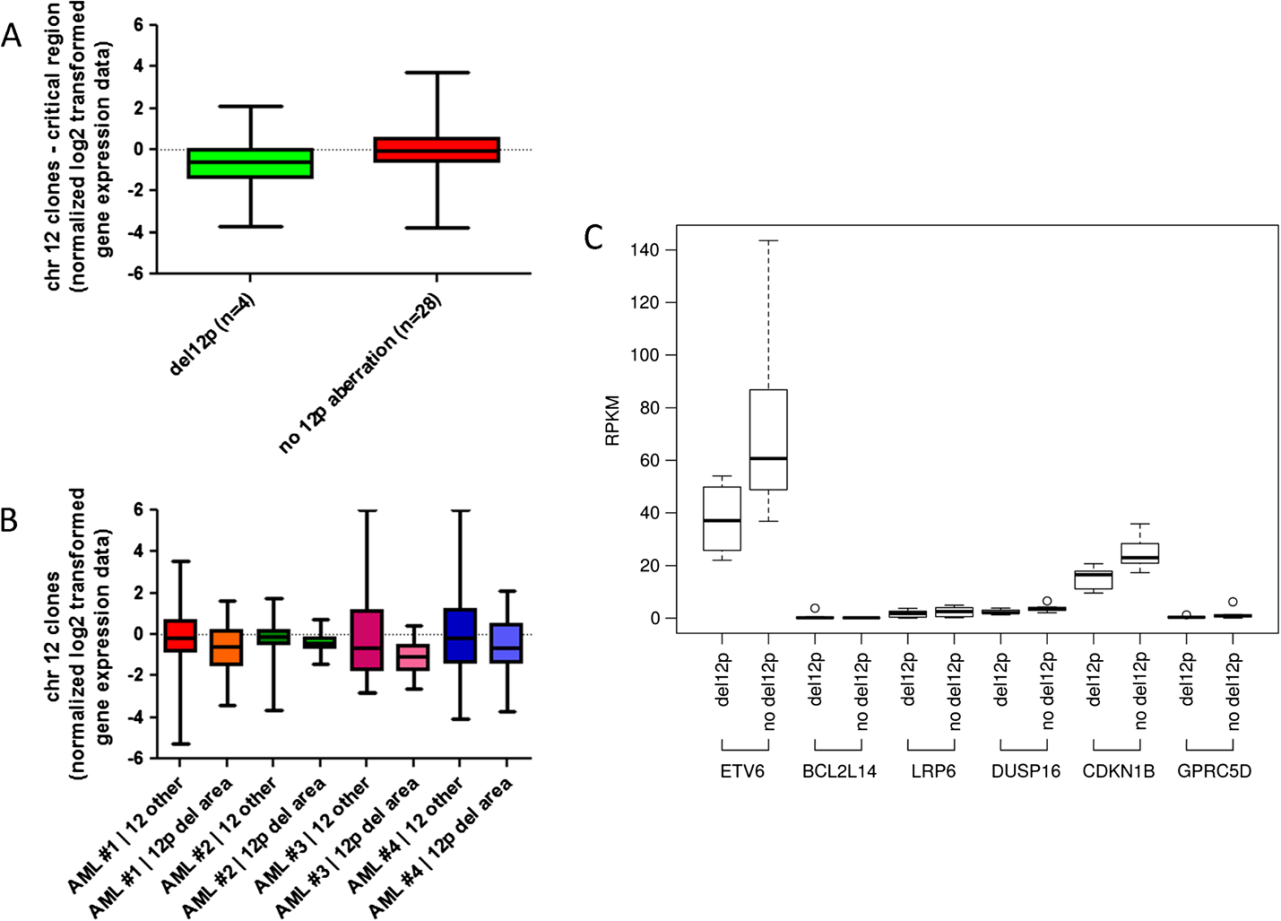
**12p13 deletion-associated gene dosage effect. A**: Comparison of CK-AML with (n = 4) and without 12p13 deletion (n = 28). The box plot shows the expression values for the genes located in the critical 12p region comprising the following genes: *ETV6, BCL2L14, LRP6, DUSP16*, *CDKN1B* and *GPRC5D* (normalized log2 transformed gene expression values). Cases with a 12p13 deletion show a significantly lower expression level for the deleted genes (p < 0.001, unpaired *t*-test). **B**: Comparison of the clones located within the critical region and clones located in unaltered chromosome 12 regions shows a significantly lower expression for the 12p critical region clones in AML cases with a 12p13 deletion (p = 0.041, ANOVA one-way analysis of variance). **C**: Comparison of CK-AML cases with (n = 8) and without 12p13 deletion (n = 8). The box plot shows the RPKM values of RNAseq data representing the expression level of the genes *ETV6, BCL2L14, LRP6, DUSP16, CDKN1B* and *GPRC5D*. Cases with a 12p13 deletion show a significantly lower expression level for *ETV6* (p < 0.03, unpaired *t*-test) and *CDKN1B* (p < 0.003, unpaired *t*-test). *BCL2L14, LRP6, DUSP16* and *GPRC5D* show no or very low expression levels. All data represented here were obtained from the TCGA network (http://cancergenome.nih.gov).

As we showed that gene dosages of *ETV6* and *CDKN1B* were reduced to half the normal level in CK-AML samples with 12p13 deletion, we were interested to determine whether this is reflected on the transcriptional level. Therefore, we first evaluated the gene dosage effect based on our previously published gene expression profiling [23, 24]. Expression and genomic data were available for four of our own patients with CK-AML and deletion in 12p13 and for 28 cases without deletion in 12p13. The four CK-AML patients with deletion in 12p13 showed a significantly lower expression level for the deleted genes than CK-AML without deletion in 12p13 (p < 0.001, unpaired *t*-test, Figure 5A). Within the four cases with 12p13 deletion we compared the expression level of the genes located in the critical region with those on chromosome 12 outside the deleted region and found a lower expression level for the deleted genes (Figure 5B).

In addition, we screened the publicly available data of The Cancer Genome Atlas (TCGA, http://cancergenome.nih.gov/) for CK-AML patients with and without deletion in 12p13. We compared eight cases of CK-AML with deletion in 12p13 and eight cases of CK-AML without deletion in 12p13. *ETV6* and *CDKN1B* are expressed in CK-AML with and without deletion in 12p13 (*ETV6*/del12p: 37.68 ± 12.95 RPKM, *ETV6*/no del12p: 71.63 ± 34.43 RPKM; *CDKN1B*/del12p: 15.19 ± 4.06 RPKM, *CDKN1B*/no del12p: 24.73 ± 5.86 RPKM). Notably, the expression level of *ETV6* and *CDKN1B* decreased significantly by 0.53-fold (p < 0.03) and 0.62-fold (p < 0.003), respectively, in cases with 12p13 deletions compared to those without 12p13 deletions. The other candidate genes located within the minimal deleted region (*BCL2L14, LRP6, DUSP16* and *GPRC5D*) showed no or very low expression levels irrespective of their copy number status (0.13 – 3.72 RPKM) (see Figure 5C).

## Discussion

12p13 deletions are common in a broad spectrum of hematological malignancies, notably in myelodysplastic syndrome (MDS) with monosomy 7 [25] and CK-AML [8–10]. *ETV6* and *CDKN1B* are the candidate tumor suppressor genes within the region 12p13 [12, 13]. Other candidate genes within the minimal deleted region like *BCL2L14*, *LRP6*, *DUSP16* and *GPRC5D* may also play a role in tumorigenesis and leukemogenesis.

However, we now demonstrate according to the expression data provided by TCGA in confirmation of data published by Haferlach *et al*, that these genes do not show any expression or very low expression in blood or bone marrow cells of AML patients [26]. Likewise, using the platform HemaExplorer (http://servers.binf.ku.dk/hemaexplorer/), they show a low expression in normal hematopoietic stem and progenitor cells, compared to the expression level of *ETV6* (5- to 40-fold higher) and *CDKN1B* (3- to 20-fold higher) in these cells [26]. It seems unlikely that they play a major role in leukemogenesis (see Figure 5C). Recent data suggest that the function of some tumor suppressor genes can be disrupted solely by haploinsufficiency leading to reduced gene dosage which might be sufficient to contribute to tumorigenesis [27–29]. Popular examples are *TP53, PTEN, NPM1*, *NF1* and *RPS14* [27, 29]. Likewise, for the genes *APC, ATM, BRCA1/2* and *RB* haploinsufficiency contributes to tumorigenesis [28]. Haploinsufficiency of *CDKN1B* is strongly implicated in numerous cancer types, the vast majority of lymphatic origin [16, 17]. *CDKN1B* heterozygous mice are predisposed to tumors in multiple tissues [30]. *ETV6* and *CDKN1B* are known to be inactivated mostly by monoallelic deletions. Yet, the mode of inactivation has not been investigated in detail in CK-AML with a high frequency of 12p13 deletions.

In our study, 12p13 deletions, mostly small interstitial deletions, were present in nearly half (49%) of the analyzed CK-AML patients according to karyotype. In 20 of 43 patients (47%), losses of 12p13 could be identified by genomic profiling, of those 13 small interstitial deletions (see Additional file 1: Table S1). The high frequency may be explained by high-resolution arrays used in this study. In previous studies based on karyotyping or SNP arrays of lower resolution, the frequency of 12p13 deletions in CK-AML was 27% [31] and 18% [10], respectively. However, it cannot be excluded that the rather high frequency in our study is due to the relatively small size of our cohort. The minimal deleted region in our cohort spanned 1.43 Mb and included *CDKN1B* and the 3′UTR of *ETV6* (see Figure 1). In one patient (#48) the distal breakpoint is located within a region between exon 8 and the 3′UTR of the *ETV6* gene*.* As the end of the ETS DNA-binding domain and the highly conserved polyadenylation signal lay within the deleted region and differential polyadenylation of the 3′UTR of *ETV6* plays a major role in posttranscriptional modification [32], we assume that *ETV6* is contained in the minimal deleted region.

*ETV6* and *CDKN1B* mutations have never been investigated specifically in CK-AML. Somatic *ETV6* mutations are rare events in newly diagnosed AML [33], AML-M0 [34] and MDS [35]. We detected one heterozygous frameshift mutation among 56 patients (see Figure 3, Additional file 1: Table S1). Our results thereby confirm the low rate of *ETV6* mutations in the distinct subgroup of CK-AML. The detected frameshift mutation lies within the N-terminal homodimerization domain leading to a complete loss of the DNA-binding ETS domain. The vast majority of all reported *ETV6* mutations results in inactivation of one *ETV6* allele which is consistent with haploinsufficiency as the underlying mechanism. There was no typical driver alteration like *TP53* or *FLT3* mutations and no typical cytogenetic aberrations of CK-AML like del(5q), -7/del(7q), or del(17p) present in this patient, which could provide a proliferative advantage or even initiate leukemogenesis. This might strengthen the role of this *ETV6* mutation as a driver rather than a passenger mutation. Unfortunately, no fibroblast DNA was available to prove whether the *ETV6* mutation was of germline origin. However, this seems to be unlikely as *ETV6* is a critical regulator in the survival of multiple cell types during early embryonic development and *ETV6* knockout mice are embryonically lethal [36].

According to the literature, *CDKN1B* mutations have been previously reported in childhood leukemia [37] and rarely in T-cell prolymphocytic leukemia [16] as well as in adult T-cell leukemia [38]. We did not identify a mutation in the *CDKN1B* gene in our analyzed patient cohort (see Additional file 1: Table S1).

As expected, in more than half of the analyzed patients, *TP53* mutations were detected (see Additional file 2: Table S2). However, the frequency of 12p13 deletions did not differ between patients with and without a monoallelic or biallelic alteration of *TP53* (45.5% versus 45.7%, p = 0.99).

One mechanism of haploinsufficiency is increased methylation leading to reduced gene dosage due to transcriptional silencing. For *ETV6*, increased methylation was suggested as a possible mechanism since decreased *ETV6* protein expression was reported in AML patients [33]. Hypermethylation of *CDKN1B* was excluded in not further defined AML and MDS, but found in the lymphoblast-like cell line Raji [39]. We demonstrate here that the CpG islands within the *ETV6* and *CDKN1B* 5′UTRs are not hypermethylated in CK-AML (see Figure 4). To our knowledge, this is the first report that excludes 5′UTR methylation leading to *ETV6* and *CDKN1B* inactivation in CK-AML and supports haploinsufficiency by heterozygous deletions as mode of inactivation.

We show by reanalysis of our previously published gene expression profiling [23, 24] of patients with CK-AML and deletion in 12p13 a significantly lower expression level for the deleted genes (p < 0.001) compared to a group of CK-AML patients without 12p13 alteration (see Figure 5A + B). Furthermore, we screened the publicly available data of TCGA and demonstrate that the other candidate genes located within the minimal deleted region (*BCL2L14, LRP6, DUSP16* and *GPRC5D*) show no or very low expression levels irrespective of their copy number. In contrast, *ETV6* and *CDKN1B* are expressed in CK-AML and their expression levels significantly decreased in cases with 12p13 deletions (see Figure 5C). Thus, *ETV6* and *CDKN1B* are the sole genes within the minimal deleted region with expression levels reduced to approximately half. These data strongly support our hypothesis that haploinsufficiency is the underlying mechanism of inactivation of *ETV6* and *CDKN1B*.

## Conclusion

In summary, we demonstrate that the putative tumor suppressor genes *ETV6* and *CDKN1B* are frequently inactivated by loss of one copy, most frequently by small deletions of 12p13, in CK-AML. The expression level of *ETV6* and *CDKN1B* is significantly decreased in cases with 12p13 deletions whereas the other potential candidate genes within the minimal deleted region do not show any or very low expression irrespective of their copy number status. Mutations and hypermethylation as mode of inactivation were largely excluded. It is possible that the genomic complexity leads to acquisition of the deletion in 12p13. Future studies investigating clonal evolution should clarify, whether haploinsufficiency of both genes may cooperate early in the process of leukemic transformation by disordering key processes of differentiation and proliferation and whether they may also play a critical role in the induction of chromosomal instability finally resulting in the development of clones with complex karyotypes.

## Methods

### Patients

79 patients with CK-AML were analyzed (see Additional file 1: Table S1). Complex karyotype was defined by the presence of at least three chromosomal abnormalities in the absence of the prognostically favorable t(8;21)(q22;q22), inv(16)(p13q22) or t(16;16)(p13;q22) and t(15;17)(q22;q12). The diagnosis of AML was made according to the French-American-British Cooperative Group criteria. The karyotypes were described according to the International System for Human Cytogenetic Nomenclature (ISCN) (2013) [40]. DNA for analysis was extracted from bone marrow or peripheral blood-derived cell pellets stored at -196°C in liquid nitrogen and from methanol/acetic acid fixed cells stored at -80°C, using the Qiagen QIAamp® DNA Micro/Midi Kit (Qiagen, Hilden, Germany).

All patients signed informed consent forms, and the project was approved by the Ethics Committee of Hannover Medical School (approval no. 2899 of 20.06.2011). The research has been conducted in compliance with the Helsinki declaration.

### Array-based genomic profiling

ArrayCGH using the 2.8 k and/or the 8.0 k platform and SNP analyses using Affymetrix GeneChip Human Mapping 250 K Array (Affymetrix, Santa Clara, California) and/or Genome-Wide Human SNP 6.0 Array (Affymetrix, Santa Clara, California) were performed as previously described by Rücker *et al* [41]. Results obtained from BAC/PAC arrayCGH are given in Additional file 3: Table S3. In brief, cut-off levels for gains and losses were determined for each individual experiment. After computing the ratios from dye-swap hybridization and subsequent normalization, an individual set of balanced clones for each experiment was used to calculate the mean and standard deviations. The cut-off levels were defined as mean plus/minus three times the standard deviation. Frequently affected regions recently detected as copy number polymorphisms (5q11, 7q22, 7q35, 14q32, and 15q11) were excluded from data analysis.

For arrayCGH using the Agilent Human Genome Microarray Kit 244A (Agilent Technologies, Waldbronn, Germany), a high resolution 60-mer oligonucleotide-based microarray, the procedures for DNA labeling, hybridization and washing were performed according to the manufacturer’s instructions (protocol version 6.1.) with some modifications as previously described by Praulich *et al* [42]. The slides were scanned on an Agilent Microarray Scanner and captured images were analyzed with Feature Extraction Software (v.10.7) (Agilent Technologies, Waldbronn, Germany). Data imaging and analysis were performed by the Agilent CGH Analytics software (v.5.0.14) with hg18 and Aberration Detection Method 2 (ADM-2) analysis algorithms set to specified thresholds and filter settings. All raw data from Agilent and Affymetrix are available under GEO (Gene expression omnibus, http://www.ncbi.nlm.nih.gov/geo/). Aberration summaries are archived under GSE55535. GEO accession numbers are given in Additional file 4: Table S4.

### Direct sequencing

Exons 1-8 of *ETV6* (ENSG00000139083), exons 1-2 of *CDKN1B* (ENSG00000111276), and exons 2-11 of *TP53* (ENSG00000141510) were PCR-amplified from genomic DNA using FastStart Taq DNA Polymerase (Roche, Mannheim, Germany). After purification with the magnetic bead-based CleanSEQ® system (Beckman Coulter, Krefeld, Germany), PCR fragments were sequenced in both directions using the GenomeLab™ DTCS Quick Start Kit and CEQTM 8000 Genetic Analysis System (Beckman Coulter, Krefeld, Germany). Cloning of PCR products was performed in patients with complex mutations to describe the mutations properly using the TOPO TA Cloning® Kit (Invitrogen, Karlsruhe, Germany). All mutations were described according to the nomenclature for the description of sequence variations of the Human Genome Variation Society (HGVS, http://www.hgvs.org/).

### Pyrosequencing

CpG islands were identified using CpG island searcher (http://cpgislands.usc.edu). We used the following settings: a G + C content of at least 55% GC, an observed CpG/expected CpG in excess of 0.65, and a minimum length of 500 bp. The pyrosequenced regions were also selected in the context of primer design, optimal PCR conditions and stringency.

Sodium bisulfite treatment of genomic DNA was performed using the EZ DNA Methylation Direct Kit™ (Zymo Research, Freiburg, Germany). Fragments for pyrosequencing were generated by PCR using the FastStart Taq™ DNA Polymerase Kit (Roche, Basel, Switzerland) with the following protocol: 10pmol of forward and reverse PCR primers (Metabion, Martinsried, Germany), and 0.78 units of FastStart Taq™ DNA Polymerase as well as different amounts of dNTPs and MgCl2. Cycle conditions were as follows: for fragment *ETV6* denaturation at 97°C for 7 min, touchdown for 10 cycles including denaturation at 96°C for 30 s, annealing at 58°C for 30 seconds (which was decreased by 0.5°C in each cycle) and extension at 72°C for 1 min, followed by 25 cycles at 96°C for 30 s, annealing at 53°C for 30 s and 72°C for 1 min, finished with 72°C for 7 min; for fragment *CDKN1B* denaturation at 97°C for 7 min, touchdown for 10 cycles including denaturation at 96°C for 30 s, annealing at 53°C for 30 seconds (which was decreased by 0.5°C in each cycle) and extension at 72°C for 1 min, followed by 25 cycles at 96°C for 30 s, annealing at 48°C for 30 s and 72°C for 1 min, finished with 72°C for 7 min. Primer sequences were as follows: fragETV6-fw: 5′-GYGGGTGGGAGGAGAG-3′; fragETV6-rev-biot: 5′-biotin-TTCTTCCAACATCTCTCCC-3′; fragCDKN1B-fw: 5′-GTAGGTTTGTTGGTAGTAG-3′; fragCDKN1B-rev-biot: 5′-biotin-AAAAAAAATCCATTAATTAC-3′.

For purification of biotinylated fragments, 5 to 13 μL of PCR products were added to a mixture consisting of 3 μL Streptavidin Sepharose HP™ Beads (Amersham Biosciences, Freiburg, Germany) and 47 μL binding buffer (Qiagen, Hilden, Germany). Single-stranded fragments were purified using the Vacuum Prep Tool™ (Qiagen, Hilden, Germany). Sepharose beads with the single-stranded templates attached were added to a PSQ 96 Plate Low™ (Qiagen, Hilden, Germany) containing a mix of 11 mL annealing buffer (Qiagen, Hilden, Germany) and 800 nmmol/L of the corresponding sequencing primers (Metabion, Martinsried, Germany) with following sequences: fragETV6-seq: 5′-GATTTG TAGATTT-3′; fragCDKN1B-seq: 5′-GATTAGTTAATTTTT-3′.

Pyrosequencing was performed in a PyroMark MD™ System (Qiagen, Hilden, Germany) with the PyroGold SQA™ Reagent Kit (Qiagen, Hilden, Germany) containing nucleotides and prepared mixtures with enzymes or substrates. For pyrogram exposure including CpG-site methylation calculation, the Pyro Q-CpG™ Software (Biotage, Uppsala, Sweden; Version 1.0.9.) was applied. Only pyrograms including sharp peaks with satisfactory heights for each injected nucleotide of interest and without peaks for unsuccessful bisulfite treatment or background controls were considered.

### Gene expression profiling analysis

Based on our previously published gene expression profiling [23, 24], we evaluated a 12p deletion associated gene dosage effect of our own patients by comparing complex karyotype cases with 12p13 deletion (n = 4) and cases without (n = 28). In brief, normalized log2 transformed gene expression levels of the minimally deleted region were averaged and compared to the average unaltered chromosome 12 regions.

SNP level3 datasets (archive: broad.mit.edu_LAML.Genome_Wide_SNP_6.Level_3.25.2004.0) and RNAseq level3 datasets (archive: bcgsc.ca_LAML.IlluminaGA_RNASeq. Level_3.1.7.0) of AML patients were obtained from the TCGA Research Network (http://cancergenome.nih.gov/). Visualization and selection of the SNP data was done using the Integrative Genomics Viewer [43, 44]. The RPKM values (RPKM = (10^9 * C)/(N * L); C = Number of reads mapped to a gene, N = Total mapped reads in the experiment, L = exon length in base-pairs for a gene) of the corresponding gene-quantification files were extracted and analyzed on gene level applying the *t*-test program of the statistical program R, version 3.1.1 (http://www.R-project.org/) [45]. Accordingly, box plots were made using the boxplot function of R.

### Statistical analysis

We used the two-tailed Fisher’s exact test, Kruskal-Wallis one-way analysis of variance, the unpaired *t*-test and the ANOVA one-way analysis of variance. An effect was considered significant if the p value was <0.05.

## Electronic supplementary material

Additional file 1: Table S1: Karyotypes, genomic profiling, sequencing results and methylation analyses of the patient cohort. Karyotypes of the 79 patients with CK-AML investigated in this study (classified according to ISCN recommendations), results obtained by genomic profiling, mutation analyses of *ETV6* and *CDKN1B* (mutations are named according to HGVS (http://www.hgvs.org/)), and mean methylation levels. (XLS 43 KB)

Additional file 2: Table S2: Overview of *TP53* mutations. *TP53* mutation status of the 79 patients investigated in this study, mutations are named according to HGVS (http://www.hgvs.org/). FISH results with a probe for the locus 17p13 (TP53) are also shown if available. (XLS 26 KB)

Additional file 3: Table S3: Normalized log2 fluorescence ratio of six CK-AML samples obtained from 2.8/8.0 k BAC/PAC arrays. Cut-off levels for gains and losses were determined for each individual experiment. After computing the ratios from dye-swap hybridization and subsequent normalization, an individual set of balanced clones for each experiment was used to calculate the mean and standard deviations. The cutoff levels were defined as mean plus/minus three times the standard deviation. Frequently affected regions recently detected as copy number polymorphisms (5q11, 7q22, 7q35, 14q32, and 15q11) were excluded from data analysis. (XLSX 1 MB)

Additional file 4: Table S4: List of GEO accession numbers. List of GEO accession numbers for all samples run on the Agilent/Affymetrix platform. (DOC 56 KB)

## Abbreviations

AML: Acute myeloid leukemia
CK-AML: Acute myeloid leukemia with complex karyotype
arrayCGH: Array comparative genomic hybridization
HGVS: Humane Genome Variation Society
ISCN: International System for Human Cytogenetic Nomenclature
MDS: Myelodysplastic syndrome
SNP array: Single-nucleotide polymorphism array
TCGA: The cancer genome atlas.
