# Supplementary material for: Haploinsufficiency of ETV6 and CDKN1B in patients with acute myeloid leukemia and complex karyotype
Source: BMC Genomics. 2014 Sep 11;15(1):784. doi: 10.1186/1471-2164-15-784 (PMC4168160; doi:10.1186/1471-2164-15-784)
Supplement: Supplementary file 4 — Additional file 4: Table S4: List of GEO accession numbers. List of GEO accession numbers for all samples run on the Agilent/Affymetrix platform. (DOC 56 KB) [file 12864_2014_6447_MOESM4_ESM.doc]

| **Sample name** | **Array-name** | **GEO accession** |
| --- | --- | --- |
| **Patient #2** | CK_AML [251469368056] | GSM1338774 |
| **Patient #7** | CK_AML [251469368036] | GSM1338775 |
| **Patient #9** | CK_AML [251469368038] | GSM1338776 |
| **Patient #12** | CK_AML [251469368058] | GSM1338777 |
| **Patient #13** | CK_AML [251469368060] | GSM1338778 |
| **Patient #16** | CK_AML [251469368084] | GSM1338779 |
| **Patient #18** | CK_AML [251469368059] | GSM1338780 |
| **Patient #20** | CK_AML [251469368057] | GSM1338781 |
| **Patient #21** | CK_AML [251469368085] | GSM1338782 |
| **Patient #22** | CK_AML [251469368062] | GSM1338783 |
| **Patient #27** | CK_AML [251469368052] | GSM1338784 |
| **Patient #31** | CK_AML [251469368053] | GSM1338785 |
| **Patient #34** | CK_AML [251469368063] | GSM1338786 |
| **Patient #35** | CK_AML [251469368061] | GSM1338787 |
| **Patient #36** | CK_AML [251469368051] | GSM1338788 |
| **Patient #37** | CK_AML [251469368054] | GSM1338789 |
| **Patient #39** | CK_AML [251469368037] | GSM1338790 |
| **Patient #40** | CK_AML [251469368065] | GSM1338791 |
| **Patient #41** | CK_AML [251469368064] | GSM1338792 |
| **Patient #48** | CK_AML [251469368050] | GSM1338793 |
| **Patient #57** | CK_AML [UPN88] | GSM850818 |
| **Patient #58** | CK_AML [UPN96] | GSM850821 |
| **Patient #59** | CK_AML [UPN37] | GSM850790 |
| **Patient #60** | CK_AML [UPN156] | 8.0k_BAC-array/suppl. Table 1 |
| **Patient #61** | CK_AML [UPN111] | 8.0k_BAC-array/suppl. Table 1 |
| **Patient #62** | CK_AML [UPN84] | GSM850814 |
| **Patient #63** | CK_AML [UPN174] | GSM850749 |
| **Patient #64** | CK_AML [UPN134] | GSM850734 |
| **Patient #65** | CK_AML [UPN212] | GSM850764 |
| **Patient #66** | CK_AML [UPN6] | GSM850805 |
| **Patient #67** | CK_AML [UPN136] | GSM850736 |
| **Patient #68** | CK_AML [UPN149] | GSM850739 |
| **Patient #69** | CK_AML [UPN225] | 8.0k_BAC-array/suppl. Table 1 |
| **Patient #70** | CK_AML [UPN49] | GSM850799 |
| **Patient #71** | CK_AML [UPN177] | 8.0k_BAC-array/suppl. Table 1 |
| **Patient #72** | CK_AML [UPN191] | GSM850754 |
| **Patient #73** | CK_AML [UPN18] | GSM850751 |
| **Patient #74** | CK_AML [UPN231] | GSM850775 |
| **Patient #75** | CK_AML [UPN161] | GSM850743 |
| **Patient #76** | CK_AML [UPN219] | GSM850768 |
| **Patient #77** | CK_AML [UPN44] | 8.0k_BAC-array/suppl. Table 1 |
| **Patient #78** | CK_AML [UPN15] | GSM850740 |
| **Patient #79** | CK_AML [UPN1] | 8.0k_BAC-array/suppl. Table 1 |
